# Piloting a Transition of Care Curriculum at Two Bi-coastal Medicine-Pediatrics Residency Programs

**DOI:** 10.7759/cureus.46418

**Published:** 2023-10-03

**Authors:** Shannon Kim, Sarah Mennito, Lori Wan

**Affiliations:** 1 Department of Pediatrics, University of California San Diego, San Diego, USA; 2 Departments of Medicine and Pediatrics, Medical University of South Carolina, Charleston, USA; 3 Departments of Medicine and Pediatrics, University of California San Diego, San Diego, USA

**Keywords:** transition of care, med-peds resident education, internal medicine, pediatrics, residency curriculum

## Abstract

Background: Lack of consistency in primary care residency training exists in transitions of care (TOC) of adolescents from pediatric to adult medicine, which can lead to conflicting or incomplete training. To fill this gap, we developed a curriculum based on the Got Transition Six Core Elements and piloted it at two bicoastal, academic Medicine-Pediatrics (Med-Peds) residency programs.

Objectives: The goals of this project are to increase resident TOC knowledge, increase transition discussion comfort and skills, and obtain feedback for curriculum improvement.

Methods: Two educational sessions were held at both institutions between 2020-2021. Of 32 potential resident participants, 26 participated in session one and 22 participated in session two. Sessions included a didactic presentation, small group activities, and a guest speaker discussing transitional experience. Electronic pre-session, post-session, and retention surveys evaluated resident knowledge, comfort, and self-reported skills of TOC. A Hybrid Type II design was used with mixed methods to evaluate curriculum effectiveness and implementation.

Results: The first and second sessions had 81% and 68% attendance, respectively. Eighty-four percent completed the pre-session survey, 65% completed the post-session survey, and 68% completed the retention survey. TOC knowledge increased by 19% overall (p<0.001). First-year residents gained the most knowledge and implementation skills. Residents participating in alternating medicine and pediatric clinics gained more knowledge than those in a combined Med-Peds clinic (p=0.001). Comfort increased for both initiating and continuing TOC discussions after the first session. Knowledge retention was not statistically significant.

Conclusion: A standardized TOC curriculum can improve resident knowledge and is easily implemented at multiple institutions. Early-in-training residents and those in alternating medicine and pediatric clinics particularly benefit.

## Introduction

Despite recommendations to initiate transition of care discussions with patients age 12-14 years [1-2], many providers do not adhere to this standard [3]. Additionally, discussions can vary by topic, frequency, and expectations [3-6]. Such variations are perpetuated by a lack of standard transition of care (TOC) education in primary care [7]. While there is an expectation for Medicine-Pediatrics (Med-Peds) residents to receive education in TOC, specifics regarding content and delivery are not standardized, which can perpetuate variations in delivery of TOC [7].

Curricula variation may result in training on only some of the Six Core Elements (6CEs) developed by Got Transition [2,8] and curricula format may include didactics [9], videos [10], small-group discussions [11], patient experiences [12], and case-based experiences [13]. Few curricula evaluate resident self-appraisal of their skills in delivering TOC discussions to patients, which limits the understanding of effectiveness [14].

Variations in transition training and execution can lead to adolescents and young adults (AYA) not undergoing a complete transition process, contributing to loss of follow-up and cessation of preventive care [15]. Lack of a primary care home can result in higher emergency department use, onset or worsening of medical conditions, and an increase in medical costs [16]. Providers who prepare AYA for transitions can help them cultivate health knowledge, healthcare autonomy, and continuity of care.

We developed a transition of care curriculum that was delivered at two Med-Peds residency programs. Our primary outcome was to assess increase in resident knowledge on transitions and improvement in self-assessment of skills in transition delivery. A secondary outcome was to evaluate curriculum content and implementation. We hypothesized that a standardized curriculum would provide residents with increased knowledge and confidence about conducting TOC discussions with their AYA patients.

This project was previously presented as a meeting poster at the Academic Internal Medicine Week 2022 (AIMW22) on April 11, 2022 in Charlotte, North Carolina.

## Materials and methods

The Med-Peds residency programs at University of California, San Diego (UCSD) and Medical University of South Carolina (MUSC) participated in this study. Each program has four residents in each post-graduate year (PGY) over four training years (PGY 1-4), for a total of 32 potential participants. At the time of the study, both UCSD and MUSC had half of their residents (eight out of 16) attend continuity clinics in separate, alternating internal medicine and pediatric clinics. The other half participated in a combined Med-Peds clinic, where they saw both pediatric and adult patients in the same clinic. 

Two educational sessions were held at each institution either in-person or via an online platform due to coronavirus disease 2019 (COVID-19) restrictions. Sessions were held in academic year 2020-2021 with the same 32 Med-Peds residents. The presentations were resident-led with guidance from Med-Peds faculty. The first session included a didactic presentation instructing on the Got Transition 6CEs. The 6CEs components include policy/guide, tracking & monitoring, readiness, planning, transfer of care, and transition completion [2]. Small group discussions and activities followed this didactic, including a review of transition case examples and development of local quality improvement projects. The second session reviewed the 6CEs and included a guest speaker who was either a young adult who transitioned his/her care or a caregiver of a child with chronic illness who transitioned. They relayed their experiences to the residents, including barriers and facilitators to transitioning, and allowed time at the end for questions and answers.

A Hybrid Type II design was used with mixed methods to evaluate the effectiveness and implementation of this curriculum via electronic surveys. Pre-session surveys evaluated resident baseline knowledge of TOC and self-assessed communication skills when discussing TOC with patients and families. Post-session surveys assessed knowledge and communication skills gained through the sessions as well as satisfaction with curriculum content and delivery. A retention survey was conducted six months after the first session and reevaluated transition knowledge and skills. Quantitative survey questions were validated through a prior transition of care curriculum project at UCSD. Qualitative survey questions were homegrown.

Gain and retention of knowledge and comfort were analyzed using the McNemar test and paired t-test. A proportional odds model was used to evaluate the correlation between the demographic data and knowledge gain. Linear regression was used to analyze questions with answer scales. To evaluate comfort gain and retention, a paired t-test determined significant changes. To analyze the correlation between demographic variables and comfort outcome, a linear regression with demographic variables as predictors was used. For qualitative feedback data, matrix analysis was used to review the data. Due to the set number of participants, achieving thematic saturation was not the goal and instead it was requesting feedback on the curriculum.

The UCSD and MUSC Institution Review Boards (IRB) both approved this study as quality improvement: UCSD Project# 201104QI and MUSC Project# 21-0721. Informed consent was obtained from the participants.

## Results

Of 32 potential participants, 26 (81%) attended the first session and 22 (68%) attended the second session. At UCSD, the first session was in-person with COVID-19 safety precautions and the second session was via an online platform. Both sessions at MUSC were held via an online platform. All sessions were 45 minutes in duration. Response rate was 84% (27/32) for the pre-session survey, 65% (21/32) for the post-session survey, and 68% (22/32) for the retention survey (Table 1).

**Table 1:** Demographics

Variable	Pre-Session Survey (n=27)	Post-Session Survey (n=21)	Retention Survey (n=22)
University of California San Diego	13	8	12
Medical University of South Carolina	14	10	10
Missing Institution	0	3	0
Post-Graduate Year 1	6	4	5
Post-Graduate Year 2	7	4	8
Post-Graduate Year 3	7	6	6
Post-Graduate Year 4	7	4	2
Post-Graduate Year “Other”	0	0	1
Missing Post-Graduate Year	0	3	0
African American	2	1	1
Asian	6	3	5
Caucasian	19	14	16
Missing Ethnicity	0	3	0
Female	18	15	15
Male	9	3	7
Missing Gender	0	3	0
Combined Medicine Pediatric Clinic	14	11	12
Alternating Medicine Pediatric Clinic	13	7	10
Missing Clinic Type	0	3	0
Number of previously attended transition educational sessions: 0	10	Not applicable	Not applicable
Number of previously attended transition educational sessions: 1	5	Not applicable	Not applicable
Number of previously attended transition educational sessions: 2	5	Not applicable	Not applicable
Number of previously attended transition educational sessions: 3	0	Not applicable	Not applicable
Number of previously attended transition educational sessions: 4+	7	Not applicable	Not applicable

**Figure 1:**
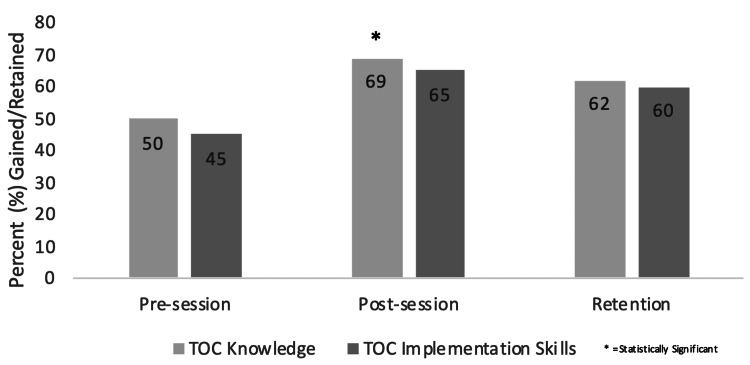
Self-Assessed Transitional Knowledge and Skills Gained and Retained TOC: transition of care

**Figure 2:**
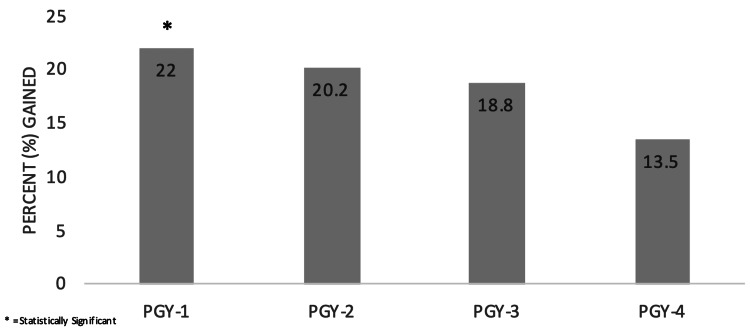
TOC Knowledge Gained From Pre-session to Post-session by Training Year TOC: transition of care, PGY: post-graduate year

Overall transition of care knowledge gained was statistically significant (p<0.001) with PGY1 residents having most gain (p=0.017) (Figures 1, 2). Residents in alternating Medicine and Pediatric continuity clinics gained more knowledge than those in a combined Med-Peds clinic (p=0.001). The greatest gain in knowledge was the age to introduce a transition of care policy (p=0.004). There was no statistical difference between training year, institution, or clinic type. Knowledge retained was not statistically significant. 

There was a statistically significant self-reported gain of transition implementation skills (p<0.001) with PGY1 residents reporting the greatest gain (p=0.011) (Figure 1). Again, residents in alternating Medicine and Pediatric clinics reported more skill improvement than those in a Med-Peds clinic (p=0.003). Increased comfort was noted for both initiating and continuing transition discussions after the first session (p=0.007 and p=0.019, respectively). There was no statistical difference between training year and clinic type for initiating transition discussions, while PGY1 residents were more comfortable continuing transition discussions after they were previously initiated (p=0.029). No statistical differences were seen between clinic type for comfort in continuing transition discussions.

Overall, the curriculum was well received by the residents. Participants liked that sessions were resident-led with faculty guidance and that the curriculum focused on aspects not previously emphasized in prior teachings, including the transition goal timeline. Residents valued the small group activities and learning from guest speakers regarding transition feasibility and barriers. Areas for improvement included having the sessions closer together in time for better continuity, incorporating sessions into the categorical education curriculum, and having the sessions earlier in the year for sooner knowledge application.

## Discussion

TOC guidance for providers, parents, and young adults have been available [17-19]. However, these resources are generally utilized post-residency and have not been consistently incorporated into residency curricula. Moreover, there is variability in the transition process between institutions and clinics [20-21]. Residency curricula incorporating transition of care knowledge and skills have been shown to be effective at single institutions [22-25]. This study showed that a standardized resident-led transition of care curriculum is effective at two institutions. 

TOC knowledge and skills show greater gain in early training years and are recommended to be implemented early in the academic year, allowing residents to increase and refine their knowledge and apply transition skills to clinical settings. Residents working in alternating medicine and pediatric clinics experienced greater knowledge gain than those in combined Med-Peds clinics. It is possible that residents in alternating clinics are less likely to have supervising faculty familiar with transition of care processes, therefore baseline knowledge prior to this curriculum was lower, allowing for greater knowledge gain. Most curricula and studies focus on patients with special healthcare needs [8,12,20-21,23,26], occur at single institutions/clinics [23,25,27-28], or evaluate transition goals for resident education as opposed to implementation [17,29-30]. This pilot curriculum executes transition education for residents caring for patients with and without special health care needs in the outpatient setting and to our knowledge, is the first resident-level transition curriculum conducted at multiple institutions. 

Limitations include small sample size and an inability to directly assess impact on patient care. However, all eligible residents were invited to participate with the majority attending each session. Lastly, the effectiveness of in-person versus online learning due to COVID-19 restrictions was not evaluated. No specific resident comments related to the curriculum noted limitations based on either format.

Curriculum modification to improve retention of knowledge and skills can include evaluations to assess transition of care implementation skills in real time and holding the sessions closer together in time. Future efforts include expanding the curriculum to other training programs including categorical Pediatrics and Internal Medicine residency programs to increase the pool of physicians trained in transitioning AYA.

## Conclusions

This educational initiative demonstrates that a standardized transition of care curriculum can improve knowledge and skills gained and is feasible for implementation at multiple institutions. Earlier implementation in training year may be more beneficial, especially for those residents in alternating Medicine and Pediatric clinics compared to those in combined clinics. Earlier implementation in the academic year may also benefit residents so that they can expand and apply their knowledge and skills during their training. Residents feel highly engaged with this peer-led structure.

These efforts can inform other programs looking to implement a standardized transition curriculum particularly in primary care training. This educational initiative also highlights the importance of being able to assess skills in real-time and providing ongoing education to improve retention. Continued modification of the curriculum is warranted through further implementation and evaluation.

## Appendices

Pre-Session Survey

1)    For your unique ID code, please enter in order:

a.     The first letter of your middle name

b.     The first letter of the month you were born

c.     The last digit of your SSN

d.     The first letter of the city you were born in

2)    Which year of training are you in?

a.    PGY1

b.    PGY2

c.     PGY3

d.    PGY4

e.    Other

3)    What is the name of your residency training program?

a.     Medical University of South Carolina

b.     University of California, San Diego

4)    Which type(s) of clinic do you practice in for your continuity clinic?

a.     Combined Medicine-Pediatric clinic

b.     Alternating categorical clinics

5)    How do you identify your gender?

a.     Male

b.     Female

c.     Other

6)    How do you identify your race?

a.     Caucasian

b.     African American

c.     Hispanic

d.     Asian

e.     Other

7)    Prior to this session, how many educational meetings or talks have you attended that addressed transitions in care?

a.     Zero

b.     One

c.     Two

d.     Three

e.     Four or more

8)    On a scale of 1 to 10 (10 is most knowledgeable), my knowledge on transition of care is: (Sliding scale)

9)    On a scale of 1 to 10 (10 is most skillful), my skills in implementing the transition of care process is: (Sliding scale)

10) At what age should patients and parents be introduced to the transition policy of the clinic?  (Free text)

11) At what age should the readiness assessment first be completed? (Free text)

12) At what age should adolescents, without intellectual disability, start making SOME of their medical decisions?

a.     12-14

b.     15-17

c.     18-21

d.     21+

13) What is an ideal age that an adolescent should transfer to an adult provider or to an adult model of care if remaining with the same provider?

a.     12-14

b.     15-17

c.     18-21

d.     21+

14) What skills should providers assist their patients in developing during a transition process?  (Select all that apply.)

___ Scheduling clinic appointments         

___ Knowing medication regimen                        

___ Understanding where to seek care                            

___ Knowing and explaining medical symptoms

___ Comprehending legal aspects of medical care (e.g. DNR)

___ Building patient decision making

___ Understanding health insurance

___ Fostering patient autonomy

15) Should transition of specialty care be assessed prior to transfer to an adult physician or should it be delayed to allow the new adult primary to lead the referral process?

a.     Prior to transfer

b.     After transfer

16) True or False: The readiness assessment is a tool used only in the year of transfer to make sure the patient is ready to change to an adult provider or an adult model of care.

a.     True

b.     False

17) On a scale of 1 to 10 (10 is most comfortable), how comfortable are you in initiating a discussion about transition of care? (Sliding scale)

18) On a scale of 1 to 10 (10 is most comfortable), how comfortable are you in speaking with an adolescent about transition of care after the initial conversation about transitions was completed at a prior visit? (Sliding scale)

19) On a scale of 1 to 10 (10 is most knowledgeable), how knowledgeable is your understanding on different types of health insurance can impact access to care during the transition process? (Sliding scale)

20) Name 3 barriers that you anticipate will prevent you from successfully transitioning your patients?  (Free text)

Post-Session Survey

1)    For your unique ID code, please enter in order:

a.     The first letter of your middle name

b.     The first letter of the month you were born

c.     The last digit of your SSN

d.     The first letter of the city you were born in

2)    Were you able to attend the Medicine-Pediatric transition of care educational session in September 202?

a.    Yes

b.    No

3)    On a scale of 1 to 10 (10 is most knowledgeable), my knowledge on transition of care is now: (Sliding scale)

4)    On a scale of 1 to 10 (10 is most skillful), my skills in implementing the transition of care process is now: (Sliding scale)

5)    At what age should patients and parents be introduced to the transition policy of the clinic?  (Free text)

6)    At what age should the readiness assessment first be completed? (Free text)

7)    At what age should adolescents, without intellectual disability, start making SOME of their medical decisions?

a.     12-14

b.     15-17

c.     18-21

d.     21+

8)    What is an ideal age that an adolescent should transfer to an adult provider or to an adult model of care if remaining with the same provider?

a.     12-14

b.     15-17

c.     18-21

d.     21+

9)    What skills should providers assist their patients in developing during a transition process?  (Select all that apply.)

___ Scheduling clinic appointments         

___ Knowing medication regimen                        

___ Understanding where to seek care                            

___ Knowing and explaining medical symptoms

___ Comprehending legal aspects of medical care (e.g. DNR)

___ Building patient decision making

___ Understanding health insurance

___ Fostering patient autonomy

10) Should transition of specialty care be assessed prior to transfer to an adult physician or should it be delayed to allow the new adult primary to lead the referral process?

a.     Prior to transfer

b.     After transfer

11) True or False: The readiness assessment is a tool used only in the year of transfer to make sure the patient is ready to change to an adult provider or an adult model of care.

a.     True

b.     False

12) On a scale of 1 to 10 (10 is most comfortable), how comfortable are you now in initiating a discussion about transition of care? (Sliding scale)

13) On a scale of 1 to 10 (10 is most comfortable), how comfortable are you now in speaking with an adolescent about transition of care after the initial conversation about transitions was completed at a prior visit? (Sliding scale)

14) On a scale of 1 to 10 (10 is most knowledgeable), how knowledgeable is your understanding on different types of health insurance can impact access to care during the transition process? (Sliding scale)

15) What aspects of the teaching session on transitions of care in September 2020 did you think were effective?  Please be specific.  If you did not attend this session, please write “N/A.”  (Free text)

16) How can we improve this teaching session on transitions of care in the future?  If you did not attend this session, please write “N/A.”  (Free text)

17) Name 3 transition of care topics that you would like to learn more about in the future.  (Free text)

Retention Survey

1)    For your unique ID code, please enter in order:

a.     The first letter of your middle name

b.     The first letter of the month you were born

c.     The last digit of your SSN

d.     The first letter of the city you were born in

2)    Which year of training are you in?

a.    PGY1

b.    PGY2

c.     PGY3

d.    PGY4

e.    Other

3)    What is the name of your residency training program?

a.     Medical University of South Carolina

b.     University of California, San Diego

4)    How do you identify your gender?

a.     Male

b.     Female

c.     Other

5)    How do you identify your race?

a.     Caucasian

b.     African American

c.     Hispanic

d.     Asian

e.     Other

6)    Which clinic model do you practice in your continuity clinic?

a.     Combined Medicine-Pediatric clinic

b.     Alternating categorical clinics

7)    Prior to this session, how many educational meetings or talks have you attended that addressed transitions in care?

a.     Zero

b.     One

c.     Two

d.     Three

e.     Four or more

8)    Which transition of care sessions, as part of your residency program, did you attend in 2020-2021?

a.     September 2020 session

b.     January/February 2021 session

c.     Both sessions (September 2020 AND January/February 2021)

9)    On a scale of 1 to 10 (10 is most knowledgeable), my knowledge on transition of care is now: (Sliding scale)

10) On a scale of 1 to 10 (10 is most skillful), my skills in implementing the transition of care process is now: (Sliding scale)

11) At what age should patients and parents be introduced to the transition policy of the clinic?  

a.     10 years old

b.     11 years old

c.     12 years old

d.     13 years old

e.     14 years old

f.      15 years old

g.     16 years old

h.     17 years old

i.      18 years old

12) At what age should the readiness assessment first be completed?

a.     10 years old

b.     11 years old

c.     12 years old

d.     13 years old

e.     14 years old

f.      15 years old

g.     16 years old

h.     17 years old

i.      18 years old

13) At what age should adolescents, without intellectual disability, start making SOME of their medical decisions?

a.     12-14

b.     15-17

c.     18-21

d.     21+

14) What is an ideal age that an adolescent should transfer to an adult provider or to an adult model of care if remaining with the same provider?

a.     12-14

b.     15-17

c.     18-21

d.     21+

15) What skills should providers assist their patients in developing during a transition process?  (Select all that apply.)

___ Scheduling clinic appointments         

___ Knowing medication regimen                        

___ Understanding where to seek care                            

___ Knowing and explaining medical symptoms

___ Comprehending legal aspects of medical care (e.g. DNR)

___ Building patient decision making

___ Understanding health insurance

___ Fostering patient autonomy

16) Should transition of specialty care be assessed prior to transfer to an adult physician or should it be delayed to allow the new adult primary to lead the referral process?

a.     Prior to transfer

b.     After transfer

17) True or False: The readiness assessment is a tool used only in the year of transfer to make sure the patient is ready to change to an adult provider or an adult model of care.

a.     True

b.     False

18) On a scale of 1 to 10 (10 is most comfortable), how comfortable are you now in initiating a discussion about transition of care? (Sliding scale)

19) On a scale of 1 to 10 (10 is most comfortable), how comfortable are you now in speaking with an adolescent about transition of care after the initial conversation about transitions was completed at a prior visit? (Sliding scale)

20) On a scale of 1 to 10 (10 is most knowledgeable), how knowledgeable is your understanding now on how different types of health insurance can impact access to care during the transition process? (Sliding scale)

21) What did you take away from the guest speaker’s testimonial in the January/February 2021 transition of care educational session?  (Free text)

22) What aspects of the teaching session on transitions of care in January/February 2021 did you think were effective?  Please be specific.  If you did not attend this session, please write “N/A.”  (Free text)

23) How can we improve this teaching session on transitions of care in the future?  If you did not attend this session, please write “N/A.”  (Free text)
